# Characterization of a Novel Binding Protein for Fortilin/TCTP — Component of a Defense Mechanism against Viral Infection in *Penaeus monodon*

**DOI:** 10.1371/journal.pone.0033291

**Published:** 2012-03-12

**Authors:** Tanate Panrat, Patuma Sinthujaroen, Benjamas Nupan, Warapond Wanna, Martti Tapani Tammi, Amornrat Phongdara

**Affiliations:** 1 Center for Genomics and Bioinformatics Research, Faculty of Science, Prince of Songkla University, Songkhla, Thailand; 2 Centre for Research in Biotechnology for Agriculture, Institute of Biological Sciences, University of Malaya, Kuala Lumpur, Malaysia; University of South Florida College of Medicine, United States of America

## Abstract

The Fortilin (also known as TCTP) in *Penaeus monodon* (*Pm*Fortilin) and Fortilin Binding Protein 1 (FBP1) have recently been shown to interact and to offer protection against the widespread White Spot Syndrome Virus infection. However, the mechanism is yet unknown. We investigated this interaction in detail by a number of *in silico* and *in vitro* analyses, including prediction of a binding site between *Pm*Fortilin/FBP1 and docking simulations. The basis of the modeling analyses was well-conserved *Pm*Fortilin orthologs, containing a Ca^2+^-binding domain at residues 76–110 representing a section of the helical domain, the translationally controlled tumor protein signature 1 and 2 (TCTP_1, TCTP_2) at residues 45–55 and 123–145, respectively. We found the pairs Cys59 and Cys76 formed a disulfide bond in the C-terminus of FBP1, which is a common structural feature in many exported proteins and the “x–G–K–K” pattern of the amidation site at the end of the C-terminus. This coincided with our previous work, where we found the “x–P–P–x” patterns of an antiviral peptide also to be located in the C-terminus of FBP1. The combined bioinformatics and *in vitro* results indicate that FBP1 is a transmembrane protein and FBP1 interact with N-terminal region of *Pm*Fortilin.

## Introduction

The White Spot Syndrome Virus (WSSV) is a common pathogen that causes significant production losses in the shrimp industry around the world. The virus has a wide host range in a variety of crustaceans, both freshwater and seawater species, including *penaied* shrimp, crabs, and crayfish. A WSSV infection typically causes lethargic behavior, cessation of feeding, a pink to reddish-brown discoloration, and white inclusion of calcium salts embedded in the cuticle especially inside the surface of the carapace. The moribund shrimp swim near the surface at the edge of ponds. The populations of shrimp showing these signs have high mortality rates with cumulative mortalities reaching up to 100% within three to ten days. The transmission of disease is typically via cannibalism of the sick or dying prawns and directly through contaminated water. The virus can persist and retain infectivity in seawater for four to seven days. The histopathology of WSSV infected shrimp shows severe nuclear hypertrophy, chromatin margination, and eosinophilic to large basophilic intranuclear inclusions with variable multifocal necrosis in most tissues of ectodermal and mesodermal origin [1]. The WSSV also severely damages the stomach, gills, antennal gland, heart, and eyes. The infection can be classified into two types: Type I is an acute infection that causes high mortality within two weeks in species such as *Penaeus monodon*, *Penaeus indicus*, and *Penaeus penicillatus*. Type II is latent.

Based on the morphology and the genomic structures, WSSV has been classified to the genus Whispovirus and the family Nimaviridae, The virions are enveloped, ovoid to bacilliform 276±26 nm long with a diameter of 121±9 nm, containing double-stranded circular DNA with the size ranging from 290 kbp to 305 kbp, which covers approximately 185 open reading frames, ORFs [1]–[3]. The virion envelope contains two major proteins, VP28 and VP19 of estimated sizes 28 and 19 kDa, respectively. The nucleocapsid consists of three major proteins VP26, VP24 and VP15 of estimated sizes 26, 24, and 15 kDa, respectively [4], [5]. A previous study indicates that the gene identified as ORF390 has anti-apoptotic properties, contains two putative caspase9 cleavage sites and a caspase3 cleavage site [6]. The WSSV can replicate and propagate in a number of cell types, such as haematopoietic cell cultures of freshwater crayfish, *Pacifastacus leniusculus* [7], hepatopancreas cells of *Penaeus monodon* [8] and ovarian cultures of *Marsupenaeus japonicas* [9]. The replication rate of the WSSV inside the nucleus of the haematopoietic cell cultures of freshwater crayfish is known to increase with increasing temperature [7].

Over the years, a lot of effort has been put into attempts to protect against WSSV infection in shrimp, such as the usage of random library peptides [10], RNA interference, siRNA, VP15 and VP28 genes and long dsRNA — shown to induce both a sequence-specific and sequence-independent antiviral immunity [11], injection of VP28-dsRNA alone [12], vaccination with viral structural proteins and a monoclonal antibody targeting VP28 [13].

Fortilin (also known as Translationally controlled tumor protein, TCTP) has attracted a lot of attention due to its implication in a variety of functions, i.e., control of cell cycle, cell growth and cell division [14], [15], microtubule stabilization [16], and as a growth factor for B-cells [17]. Fortilin is present in both the nucleus and cytosol [18], is inducible by serum stimulation [19] and heavy metals [20]. Fortilin has a potent anti–apoptotic function [18], [21]–[23] through binding interactions with MCL1 (Myeloid cell leukemia 1) [24] and BCL-XL (B-cell leukemia XL) [25], belonging to the anti-apoptosis BCL-2 protein family. Fortilin can also bind to Ca^2+^ [26]–[29] and thus prevent cytosolic Ca^2+^ levels from increasing and activating Ca^2+^-dependent apoptosis pathways [28]. Fortilin is thought to be a modulator of GTPase activity, acting as a molecular switch for a vast number of cellular processes in all eukaryotes [30]. Fortilin can bind to Na, K^−^ATPase and regulates the function of Na, K^−^ATPase in cooperation with Nexin6 (SNX6) [31]. The Fortilin protein is also known to be involved in growth and development, it is encoded by abundant mRNA species, and was initially characterized in mouse Ehrlich ascites tumor cells and erythroleukemia cells [15], [32]. Despite various functions, its wide range of distribution, and high level of conservation among many organisms, the distinct functions of Fortilin still remain unclear [16], [20], [26]–[28], [33].

Our group has previously identified Fortilin in *Penaeus monodon*, named *Pm*Fortilin (Accession No: AY186580.1) and shown it to have anti-apoptotic properties. This protein is well conserved, contains a Ca^2+^-binding domain, TCTP signatures similar to that of the Fortilin or TCTP genes reported in plants and animals [29]. We found it to be present at high levels during an onset of viral infection in *P. monodon* and based on a yeast two-hybrid screening assay and GST-pulldown, we further found Fortilin to interact with a previously unknown protein, named Fortilin Binding Protein 1 (FBP1) (Accession No: EU435133.1) [34]. This is a small peptide of 93 amino acids long with a molecular weight of 11 kDa, expressed solely in hemocytes and no homologs have yet been found. It was also of interest that we found transcripts of FBP1 to be up- regulated during WSSV infection with the highest level occurring at 48 h post-infection [34]. In this manuscript we report investigations into the interaction of *Pm*Fortilin with FBP1 and its function. From our results we suggest that FBP1 is a transmembrane protein and its likely function is to facilitate transport of *Pm*Fortilin across the cell membrane.

## Results

### Structural analysis of *Pm*Fortilin and FBP1

**Figure 1:**
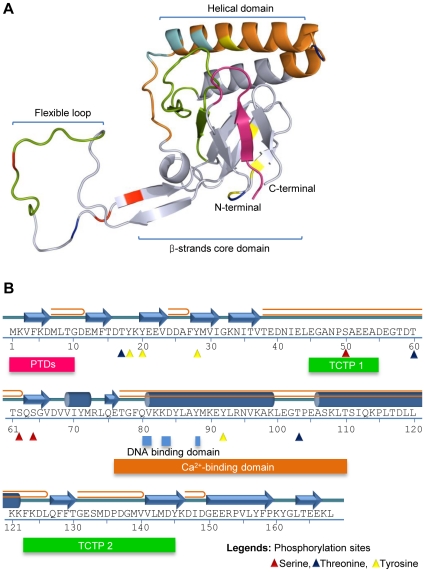
The predicted 3-D model and functional mapping of Fortilin from *Penaeus monodon*. (A) The predicted structure model of *Pm*Fortilin contains six beta hairpins, eleven strands and three helices: The helical domain (helix-helix interaction) along the residues 77–126 and a flexible loop at residues 38–62. (B) A graphical representation of the functional mapping of *Pm*Fortilin protein, was analyzed by SMART and the Motif Scan server. The following elements are shown: TCTP signatures at residues 45–55 on the flexible loop and 123–145 on the C-terminal, Ca^2+^-binding domain at residues 76–110 and DNA binding domain at residues 80–81, 83–84 and 88 also on the helical domains, serine phosphorylation sites at the residues 50, 62 and 64, threonine phosphorylation sites at the residues 17, 60 and 103, tyrosine phosphorylation sites at the residues 18, 20, 28 and 92. The ten amino acid residues at the N-terminal show high conservation with the protein transduction domains (PTDs) and may allow trans-membrane transport.

**Figure 2:**
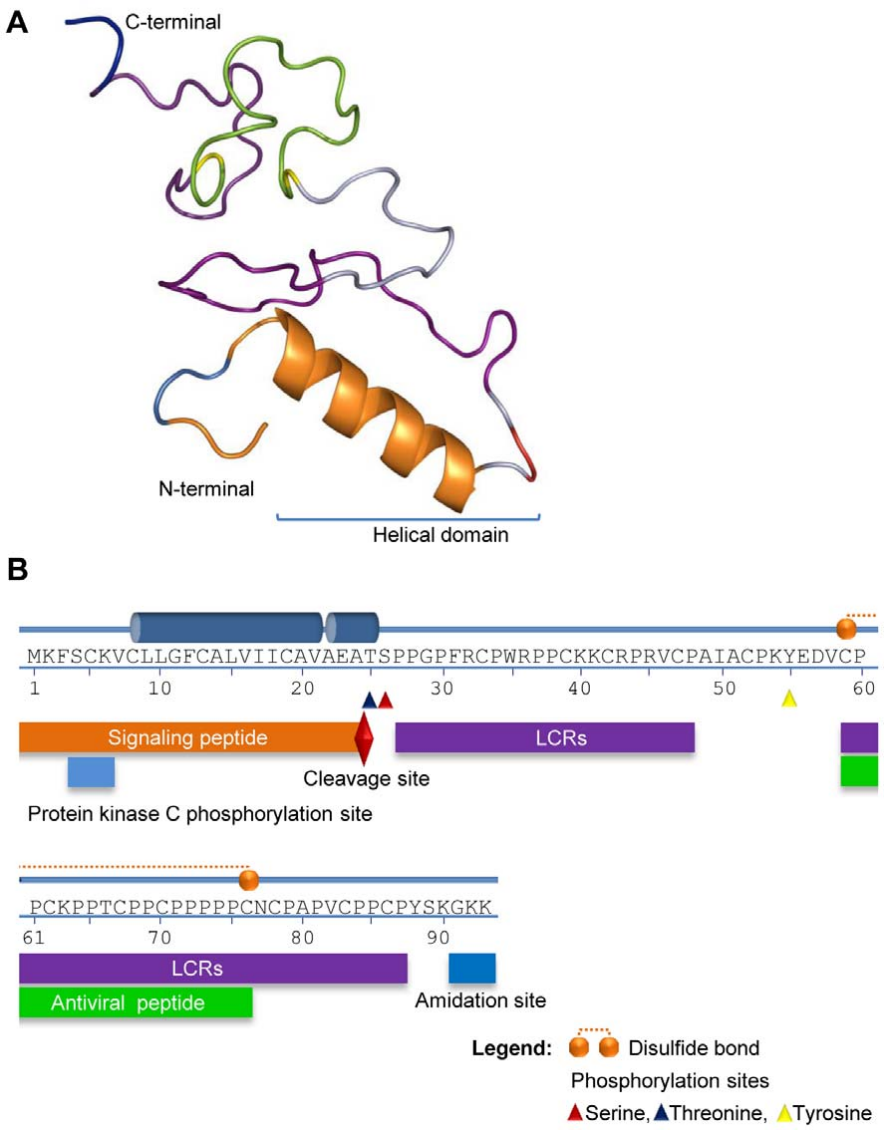
The predicted 3-D model and functional mapping of Fortilin Binding Protein 1. (A) The FBP1 contains two helical domains located at residues 8 to 21 and residues 23 to 25, forming a disulphide bond between the Cys59 and Cys76. (B) A graphical representation of the functional mapping of FBP1. The following elements are shown: Two helix domains, 16 beta turns and 12 gamma turns. A signal peptide at residues 1–24, a cleavage site at residues 22–25, with cleavage between Ala24 and Thr25. A protein kinase C phosphorylation site “[ST]–x–[RK]” at residues 4–6 at the N-terminal. An amidation pattern “x–G–[RK]–[RK]” at residues 90–93 at the C-terminal end. Two segments of compositionally biased regions or Low Complexity Regions (LCRs) at 27–48 and 59–87 and “x–P–P–x” signature sequences of antiviral peptide signatures at 59–76, serine phosphorylation site at the residue 26, threonine phosphorylation site at the residue 25, tyrosine phosphorylation site at the residue 55.

Experimentally determined structures are not available for either *Pm*Fortilin or FBP1. For this reason, we have predicted the structures by homology modeling. The best scoring 3-D structure of *Pm*Fortilin contains three sheets, six beta hairpins, two beta bulges, three helices and one helix-helix interaction. The model displays one helical domain, stretching from residue 77 to 126 and one right handed hook (RHH) of the disulphide type (Figure 1). The model of FBP1 contains two helix domains, 16 beta turns and 12 gamma turns. The two helix domains are located at residues 8 to 21 and residues 23 to 25, forming disulphide bonds between the Cys59 and Cys76 (Figure 2).

To assess the accuracy of the structure predictions, we performed a ramachandran analysis, for *Pm*Fortilin, 108 (72.50%) residues were in the most favored regions, additional allowed regions contained 33 (22.10%) residues, generously allowed regions contained seven (4.70%) residues and disallowed regions contained only a single (0.70%) residue (Figure S1). For the predicted FBP1 structure, 31 (49.20%) residues were plotted in the most favored regions, additional allowed regions contained 21 (33.30%) residues, generously allowed regions contained six (9.50%) residues and five (7.90%) residues were located in the disallowed regions (Figure S2).

*Pm*Fortilin is highly conserved; the chain A has the solution structure of human translationally controlled tumor protein, the chain A with the translationally controlled tumor associated protein p23fyp from *Schizosaccharomyces pombe*, the chains A, B, C, and D with the crystal structure of human translationally controlled tumor associated protein (hTCTP) mutant E12V, the chain A with the crystal structure of translationally controlled tumor associated protein from *Plasmodium knowlesi* and with the Fortilin structure of *Drosophila melanogaster* (PDB ID: 2HR9, 1H6Q, 3EBM, 1TXJ, and 1YZ1 respectively). A further comparison of multiple sequences reveals that *Pm*Fortilin contains conserved regions that correspond to the helical domain in the predicted 3-D structure. The amino acid residues 76–110, part of the helical domain of *Pm*Fortilin are contained within a Ca^2+^-binding domain, forming a helical domain between the H2-helix (residues 81–99) and the H3-helix (residues 106–122) producing an EF-hand structure (Figure 1). Importantly, a small section at the N-terminus (residues 1–10) contains protein transduction domains (PTDs), 1-MKVFKDMLTG-10, allowing for the delivery of active molecules into the cells through the lipid bilayer [35]–[37]. *Pm*Fortilin contains two signature patterns. The TCTP_1 and TCTP_2 at the residues 45–55 and 123–145 with the signatures “[IFAED]–[GA]–[GASF]–N–[PAK]–S–[GTA]–E–[GDEVCF]–[PAGEQV]–[DEQGAV]” and “[FLIV]–x(4)–[FLVH]–[FY]–[MIVCT]–G–E–x(4,7)–[DENP]–[GAST]–x–[LIVM]–[GAVI]–x(3)–[FYWQ]”, respectively (Table S1).

For FBP1, we were unable to find homologous sequences in the existing public databases. However, we used Motif Scan (http://myhits.isb-sib.ch/cgi-bin/motif_scan) to detect signatures. The search revealed some interesting signatures: Amino acid residues 4–6 at the N–terminal represented by “[ST]–x–[RK]” pattern at protein kinase C phosphorylation site and by “x–G–[RK]–[RK]”, which is a known amidation pattern, were found to be located at the residues 90–93 at the C-terminal end of FBP1 (Table S1). Two segments of compositionally biased regions or Low Complexity Regions (LCRs), were located at the amino acid residues 27–48 and 59–87 using SMART server (http://smart.embl-heidelberg.de) (Table S1 and S2), previously implied in the context of protein-protein interactions [38]–[40]. The residues 1–24 are likely to be a signal peptide (scored 99.00%) with a cleavage site at Ala24 and Thr25 (scored 87.30%), determined by the Signal P 3.0 server (http://www.cbs.dtu.dk/services/SignalP). It was of some interest that FBP1 contains a number of “x–P–P–x” signature sequences found in antiviral peptides. The longest such pattern is located at residues 59–76 [34]. In addition, we performed combinatorial structure analyses using GANGSTA and GANGSTA+ servers (http://agknapp.chemie.fu-berlin.de/gplus) (Table S3).

### Functional analysis of Fortilin/TCTP binding protein (FBP1)

The predicted 3-D model of FBP1 contains two alpha helix domains. Such segments are common protein structure elements that cross biological membranes. To further assess the topology and possible localization of FBP1, we utilized HMMTOP (http://www.enzim.hu/hmmtop) and TMHMM2 servers (http://www.cbs.dtu.dk/services/TMHMM). According to these predictions, the helix residues 7–26 are transmembrane segments (Table S1), the residues 1–6 that correspond to the N-terminal phosphorylation site found by Motif Scan are located in the cytosol and the residues 27–93, containing LCRs and “x–P–P–x” signature sequences, are extracellular (Figure 2B).

To find further support to the hypothesis that the FBP1 is a transmembrane protein, we transfected Sf9 cells with a plasmid containing an FBP1-GFP construct. An empty phMGFP plasmid and mock-transfected cells were used as negative controls. Confocal laser scanning microscopy was used to observe the fluorescence patterns in the cells 48 h after transfection.

**Figure 3:**
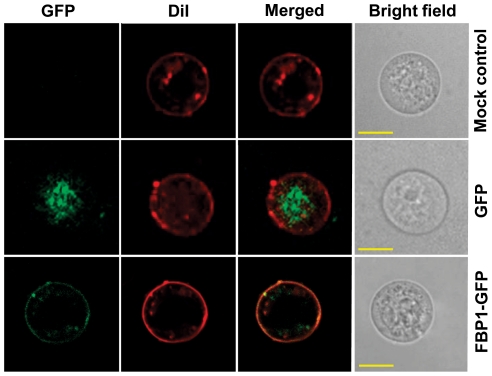
Transmembrane localization of FBP1-GFP in Sf9 cell. The cellular localization of FBP1-GFP expressed in Sf9 cell was observed using confocal laser scanning microscopy at 48 h post-transfection. The plasma membrane was stained with DiI (Invitrogen) and shows co-localization of both fluorescent (green for GFP and red for DiI) after merging. Bright field is shown in the last panel. The scale bars indicate 10 µm.

The signals in the Sf9 cells are clearly located at the plasma membrane, whereas the signals in the cells transfected only with GFP are dispersed. The mock-transfected cells show no GFP signal (Figure 3). In addition plasma membranes stained with 1,1′-Dioctadecyl-3,3,3′,3′-Tetramethylindocarbocyanine Perchlorate (DiI) confirmed the localization of FBP1 (Figure 3, lower panel).

### The Co-localization of *Pm*Fortilin and FBP1 by immunocytochemistry

**Figure 4:**
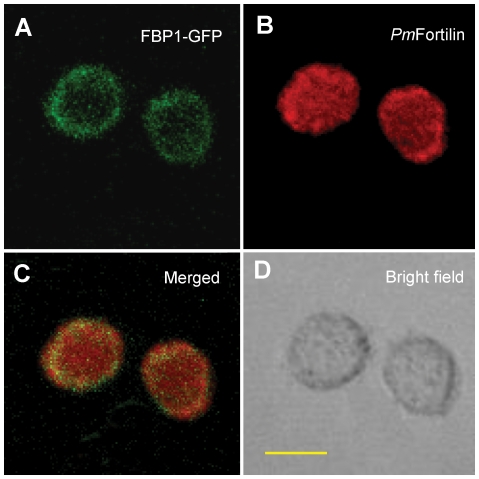
Live-cell and confocal imaging of the subcellular co-localization of GFP-FBP1 and *Pm*Fortilin. After co-transfection (48 h), the sf9 cells were fixed with paraformaldehyde and stained with rabbit anti-*Pm*Fortilin antibody, followed by Alexa Fluor 647-conjugated goat anti-rabbit IgG antibody (Invitrogen). The cells were observed with a confocal laser scanning microscope. The scale bars indicate 10 µm.

Immunofluorescence studies were performed to investigate the localization of *Pm*Fortilin in the presence of FBP1. A confocal laser scanning microscopy was used to observe the fluorescence patterns in the cells 48 h after transfection. We observed that FBP1-GFP was located at the plasma membrane (Figure 4A) while the staining with the anti-*Pm*Fortilin antibody appeared in the whole cell (Figure 4B). According to previous reports, TCTP/Fortilin was mainly localized in the cytosol and also in the nucleus [14], [18]. In this experiment, the co-localization of *Pm*Fortilin and FBP1 was detected as a yellow color only at the plasma membrane (Figure 4C).

### *Pm*Fortilin/FBP1 docking simulations and interaction analysis

**Figure 5:**
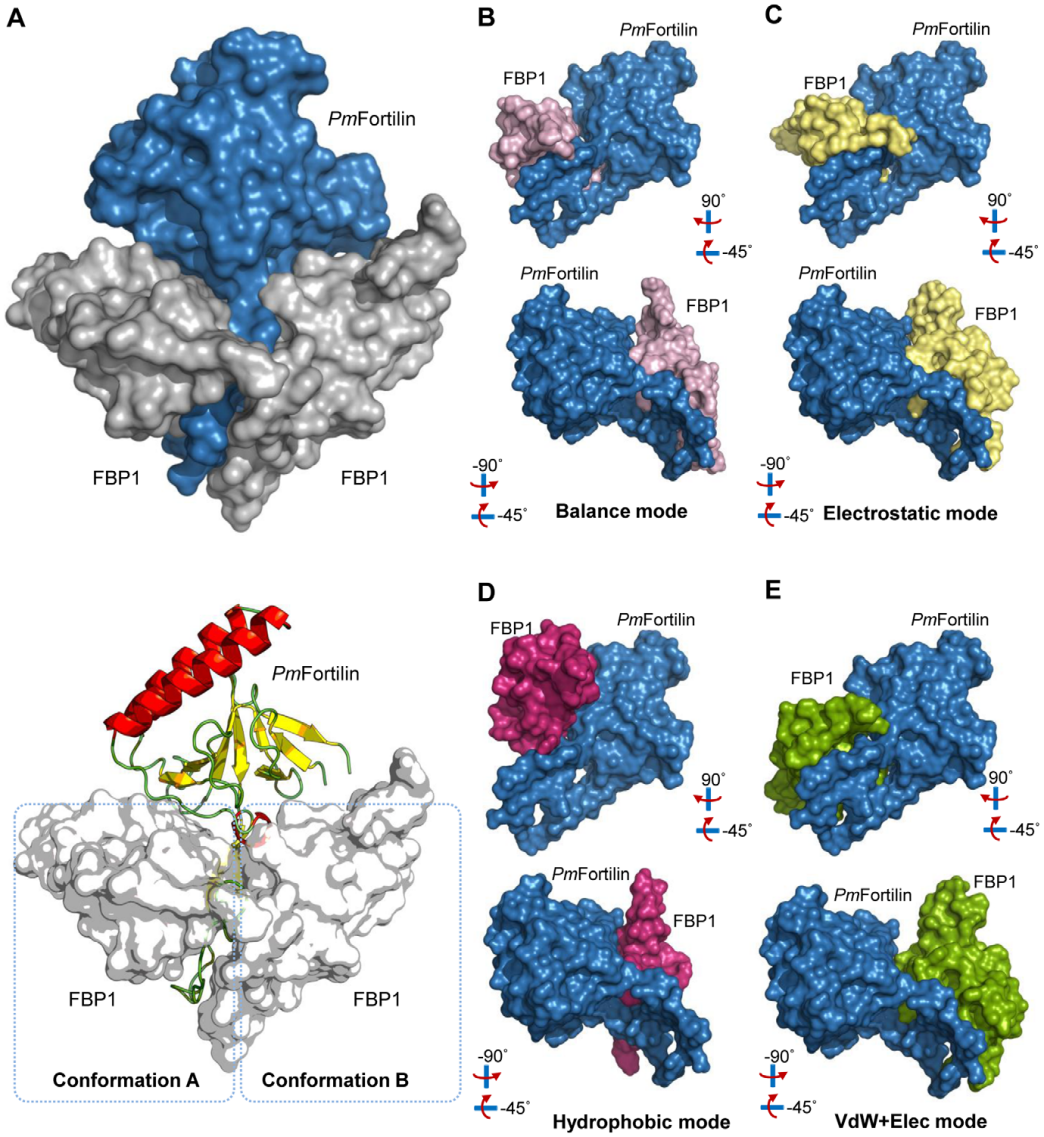
Molecular interaction models of *Pm*Fortilin/FBP1. (A top) A space-filling model representing the combination of two possible interactions of FBP1 (grey), at the opposite sides of *Pm*Fortilin (blue). (A bottom) A cartoon of the space-filling model showing the two conformations, A and B and the binding of *Pm*Fortilin to the C-terminus of FBP1. The predictions were performed with four separate modes: Balance, Electrostatic, Hydrophobic and VdW+Elec mode. (B–E) The lowest energy conformations of each of the four docking modes. The *Pm*Fortilin molecule (blue) and FBP1 (pink, yellow, magenta and green).

To further analyze the *Pm*Fortilin and FBP1 interaction in detail, we predicted the *Pm*Fortilin/FBP1 binding site residues and performed docking simulations utilizing the ClusPro 2.0 server (http://cluspro.bu.edu), including the PIPER docking software based on the Fast Fourier Transform (FTT) method [41], using FBP1 as a receptor protein and *Pm*Fortilin as a ligand. The models were predicted using four separate modes: Balance, Electrostatic, Hydrophobic and VdW+Elec mode. The short interacting elements were extracted from the best scoring interaction complex and collected to produce a refined model (Figure 5). The best, i.e. the lowest docking score of the balance mode is −993.40 Kcal/mol and highest −764.20 Kcal/mol. The average score of the docking energy center is −811.29 Kcal/mol and the lowest docking energy score is −871.40 Kcal/mol (Table S4). The electrostatic mode: Lowest docking score is −1,262.30 Kcal/mol and highest −990.60 Kcal/mol, the average of the docking energy center is −1,034.04 Kcal/mol and lowest docking energy score is −1,117.93 Kcal/mol (Table S5), the hydrophobic mode: Lowest docking score is −1,230.50 Kcal/mol and highest −951.50 Kcal/mol, the average docking energy center is −940.41 Kcal/mol and the lowest docking energy score is −1,045.73 Kcal/mol (Table S6) and the VdW+Elec mode: The lowest docking score is −387.90 Kcal/mol and highest −315.70 Kcal/mol, the average of docking energy center is −315.00 Kcal/mol and the lowest docking energy score obtained is −340.49 Kcal/mol (Table S7).

According to the docking simulations, the flexible region of *Pm*Fortilin binds to the C-terminus of FBP1. These interactions are represented in two major conformations A and B (Figure 5A). The balance mode simulation resulted in 25 separate models. The conformation A is favored over conformation B with frequencies 18/25 (72.00%) and 7/25 (28.00%) respectively (Figure 5B). Four of the top five ranking conformations are described in Table S4. The electrostatic mode yields the frequencies 17/23 (73.91%) and 6/23 (26.09%) for the conformations A and B respectively (Figure 5C), the hydrophobic mode yields the frequency 6/23 (26.09%) for conformation A and 17/23 (73.91%) for conformation B (Figure 5D) and the VdW+Elec mode yields the frequencies 22/23 (95.65%) and 1/23 (4.35%) for conformations A and B, respectively (Figure 5E). The frequencies seem to be in good agreement, except for the hydrophobic mode, where the conformation B is the most favored.

**Figure 6:**
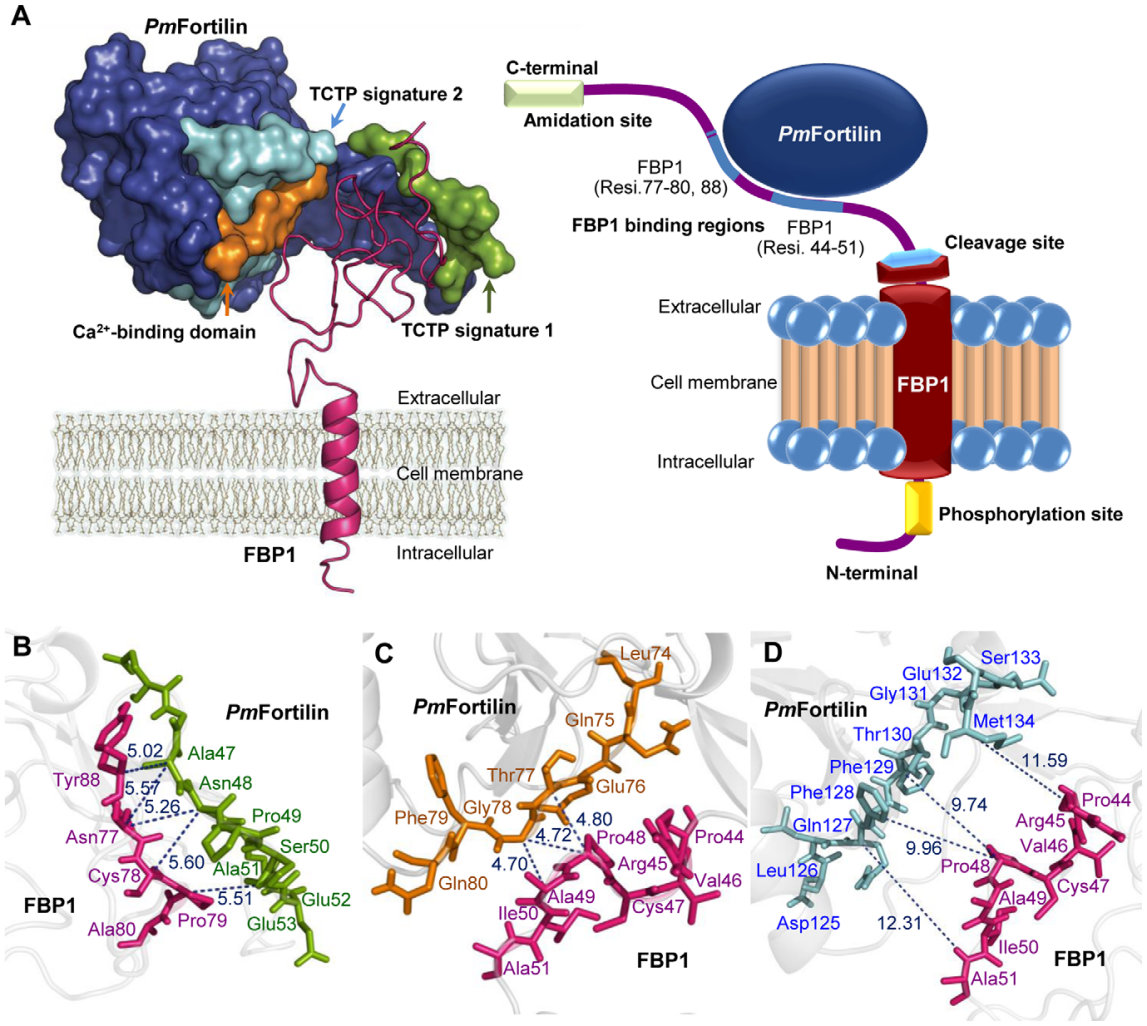
Transmembrane topology and *Pm*Fortilin/FBP1 interaction complex. (A left) The FBP1 protein integrated with the cell membrane, bound with *Pm*Fortilin. The residues 1–6 are intracellular, the residues 7–26 include the transmembrane segment and the residues 27–93 are extracellular, binding to *Pm*Fortilin. Ca^2+^-binding domain is shown in the orange color, the TCTP_1 on the flexible loop is in green, the TCTP_2 on the C-terminal **is** in light blue and other residues are in blue. (A right) A cartoon of the *Pm*Fortilin interaction complex, the FBP1 binding to *Pm*Fortilin and integration with the cell membrane; the *Pm*Fortilin (blue oval), the amidation site (light green), the cleavage site (light blue hexagon), the phosphorylation site (yellow), and the blue regions denote the binding regions of FBP1 at amino acid residues Pro44–Ala51 and Asn77–Ala80, Tyr88. This is according to the best of the interaction with the balance mode docking (probability is 72.00%). (B) The distances of amino acids in TCTP_1 interacting with the C-terminus of FBP1: Ala47 of *Pm*Fortilin and Asn77, Try88 of FBP1 and, Asn48 of *Pm*Fortilin and Asn77, Cys78 of FBP1, and Ala51 of *Pm*Fortilin and Pro79 of FBP1. (C) The distances in Angstroms between adjoining amino acids between the *Pm*Fortilin Ca^2+^-binding domain and FBP1: Thr77 of *Pm*Fortilin and Pro48 of FBP1, Gly78 of *Pm*Fortilin and Pro48, Ala49 of FBP1. (D) The distances of amino acids in the TCTP_2 interaction with the C-terminus of FBP1: Gln127 of *Pm*Fortilin and Ala51 of FBP1, Phe128–129 of *Pm*Fortilin and Pro48 of FBP1, Met134 of *Pm*Fortilin and Pro44 of FBP1.

These interactions coincide with three domains: (1) the TCTP_1 on the flexible loop of *Pm*Fortilin (Figure 6A-left; green color) with the distance scores of 5.02 and 5.57 respectively for (*Pm*Fortilin_Ala47)–(FBP1_Try88; FBP1_Asn77), 5.26 and 5.60 for (*Pm*Fortilin_Asn48)–(FBP1_Asn77; FBP1_Cys78) and 5.51 for the interaction of (*Pm*Fortilin_Ala51)–(FBP1_Pro79). (2) The predicted Ca^2+^-binding domain (Figure 6A-left; orange color) with the distance score of 4.80 for (*Pm*Fortilin_Thr77)–(FBP1_Pro48), 4.72 and 4.70 for (*Pm*Fortilin_Gly78)–(FBP1_Pro48; FBP1_Ala49). (3) The TCTP_2 on the C-terminal of *Pm*Fortilin (Figure 6A-left; light blue color) with the distance scores of 12.31 for (*Pm*Fortilin_Gln127)–(FBP1_Ala51), 9.96 for (*Pm*Fortilin_Phe128)–(FBP1_Pro48), 9.74 for (*Pm*Fortilin_Phe129)–(FBP1_Pro48), and 11.59 for (*Pm*Fortilin_Met134)–(FBP1_Pro44), according to the balance mode (Figure 6B–D).

**Figure 7:**
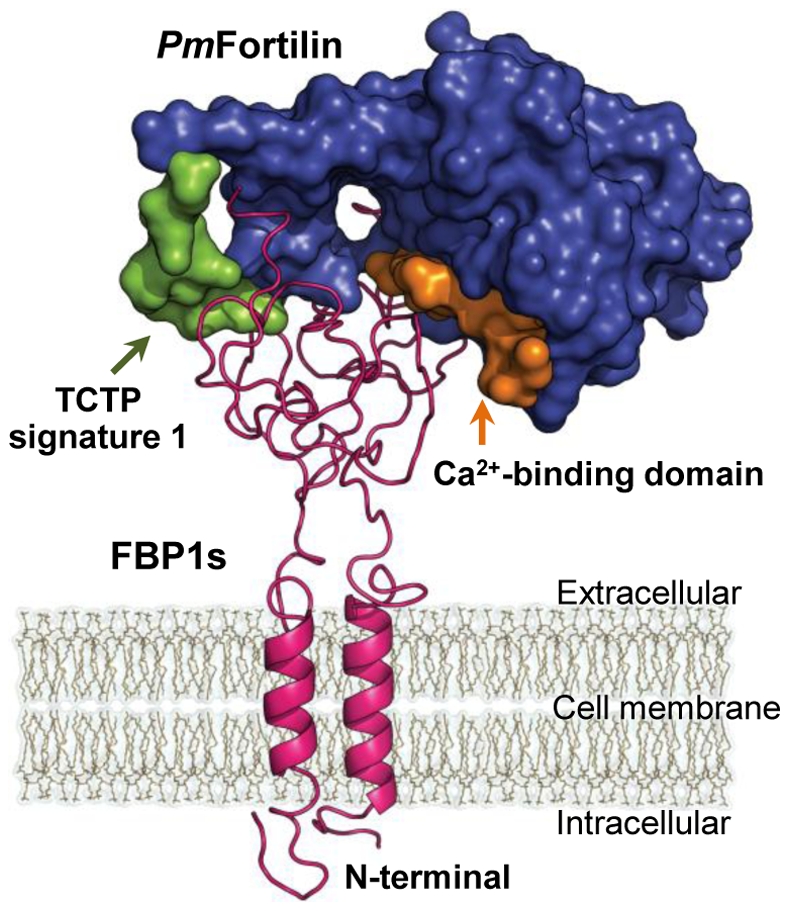
The *Pm*Fortilin/FBP1 trimer (FBP1-*Pm*Fortilin-FBP1) interaction complex. The Ca^2+^-binding domain (orange) and the TCTP_1 of the flexible loop (green). The trimer docking simulation based on the balance mode yielded the lowest docking energy score of −1,162.00 Kcal/mol, the highest −888.70 Kcal/mol and the average docking energy of −986.43 Kcal/mol.

The docking simulations predict two distinct conformations. This implies a trimer: FBP1-*Pm*Fortilin-FBP1. For this reason, we performed additional docking simulations, this time simultaneously using two FBP1s as receptors. In the trimer structure, the Ca^2+^-binding domains and the flexible loop of the *Pm*Fortilin structure interacted as in the dimer (FBP1-*Pm*Fortilin) simulations. The trimer docking simulation based on the balance mode gave the lowest docking energy score of −1,162.00 Kcal/mol and the highest −888.70 Kcal/mol, the average of docking energy being −986.43 Kcal/mol (Figure 7 and Figure S3A–D).

### Yeast two–hybrid assay – N-terminal region of *Pm*Fortilin is responsible for interaction with FBP1

**Figure 8:**
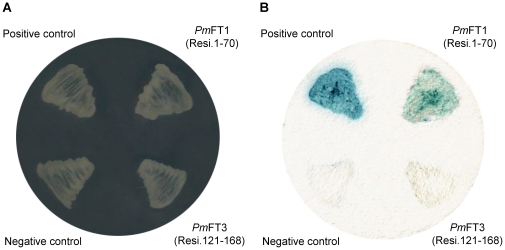
The yeast two–hybrid assay. The yeast two-hybrid interaction between *Pm*Fortilin fragments and FBP1 in *S.cerevisiae* AH109 cells. (A)YPDA control to show yeast growth. (B) β-galactosidase activity of the yeast cultures in selective medium determined by a colony-lift filter assay.

According to the docking simulations, the *Pm*Fortilin binds FBP1 at residues 37–63 located on the flexible loop of the *Pm*Fortilin N-terminus and contacts FBP1 at residues 77–88. To confirm the docking results, we sub-cloned *Pm*Fortilin in three separate fragments: *Pm*FT1 (residues 1–70), *Pm*FT2 (residues 71–120) and *Pm*FT3 (residues 121–168). The interaction of FBP1 with each of the three fragments was tested by the yeast two-hybrid assay. The yeast growth in YPDA was monitored to ensure that there were no growth defects in all recombinant clones (Figure 8A). After co-transformation, the clones that harbored the binding proteins were selected from a selective medium, at this step there were no clones found from the yeast harboring BD-*Pm*FT2+ AD-FBP1 (Table 1). This indicated that these two proteins did not interact with each other. The β-galactosidase filter assay was then used to test the yeast harboring BD-*Pm*FT1+AD-FBP1 and that harboring BD-*Pm*FT3+AD-FBP1. The result confirmed an interaction only for BD-*Pm*FT1+AD-FBP1, thereby supporting the docking results (Figure 8B). Further investigations are required to understand the binding of *Pm*Fortilin and FBP1.

**Table 1:** Summary of the growth of yeast harboring recombinant plasmids.

Yeast harboring plasmids	YPDA	Selective medium
BD-FT1 + AD-FBP1	+ + + +	+ + + +
BD-FT2 + AD-FBP1	+ + + +	−
BD-FT3 + AD-FBP1	+ + + +	+ + +

+ , the growth was observed within 3 days; −, no growth.

## Discussion

In previous report FBP1 was identified and cloned from the library by yeast two hybrid screening using *Pm*Fortilin as the bait. The interaction of these two proteins was confirmed by GST pull down [34]. Both *Pm*Fortilin and FBP1 transcripts have been show to be up regulated during a viral infection, thus implying a role in a defense mechanism against viruses. Here we try to accelerate a way to design proper biochemical experiments for the functional analysis of a novel gene product, FBP1 by using *in silico* methods. We have identified FBP1 as a membrane binding protein and analyzed its interaction with *Pm*Fortilin. The prediction was supported by the co-localization of Fortilin and FBP1 at the same position on plasma membrane. The contact sites of *Pm*Fortilin are located at amino acid residues 37–63. The latter is on the flexible loop of *Pm*Fortilin that contains the TCTP_1. The contact sites of FBP1 are located at the amino acid residues 44–51 and 77–88. The C-terminal of FBP1 is located outside of the cell membrane and contains the Cys59 and Cys76 pair forming a disulfide bond, which together with the LCRs of FBP1 and the N-terminal of *Pm*Fortilin facilitates a high-affinity binding to FBP1. The possible function of the “x–G–K–K” feature at the amidation site of FBP1 is to protect the C-terminus from being modified by proteases. Further investigation by using site directed mutagenesis at the predicted locations are required to support this conclusion.

In addition to the above analysis results, given that the signal peptides and transmembrane helixes contain hydrophobic amino acids, the existence of an N-terminal phosphorylation site and the cleavage site at Ala24 and Thr25, the results indicate that the function of FBP1 is to facilitate the transport of *Pm*Fortilin through the cell membrane. Additional docking simulations further indicate that *Pm*Fortilin can bind two FBP1s simultaneously and thus may be active as a trimer. However, further investigations are needed to decipher the detailed mechanism of transport, phosphorylation and cleavage.

## Materials and Methods

### Molecular modeling

The homology modeling for the 2-D and 3-D structures were performed with SWISS-MODEL [42] and I-TASSER [43]. The best template was automatically selected based on multiple-threading simulations and used for the structural model. Each of the predicted models was validated based on the best scoring model given by the PROCHECK software [44]. These were evaluated by ramachandran plot analysis utilizing ProFunc [45] (http://www.ebi.ac.uk/thornton-srv/databases/profunc) and PROCHECK software. The 3-D template structures were brought from the PDB database (http://swissmodel.expasy.org).

### Functional and molecular homology analysis

The GANGSTA and GANGSTA+ [46] were used for comparisons of the 3-D structures of *Pm*Fortilin and FBP1. Motif Scan [47] and SMART [48] were used for scanning signature domains of the *Pm*Fortilin and FBP1 with the default parameters, including outlier homologs and homologs of known structures, Pfam domains, signal peptides, internal repeats and intrinsic protein disorder databases.

The ProFunc server was used to identify the potential functions within the 3-D structures. The HMMTOP [49] with the ‘reliable’ mode and Baum-Welch iteration and TMHMM servers [50] were utilized to predict helical transmembrane segments and the topology with default parameters. Signal peptides and cleavage site patterns were predicted using the SignalP server [51], trained on eukaryotes. Multiple sequence alignments were constructed by using Clustal [52].

### Molecular docking simulations and analysis of the interaction model

The molecular interaction and docking simulations were performed using the ClusPro 2.0 server [53]. The interaction elements, such as the residue members, clusters and docking energy values were extracted and collected for simulation. The model of the interaction complex was assessed by the root mean square deviation (RMSD) value and the top ranking, i.e. the lowest docking energy score was used to build the construct of the interaction model. The molecular surfaces and protein structure models in the figures are built and visualized with the PyMOL [54] software on the BioSLAX suite (http://www.bioslax.com).

### Construction of recombinant FBP1-GFP and confocal microscopy

FBP1 was cloned into the phMGFP vector (Promega, catalogue no. E6421) to generate an FBP1-GFP fusion protein. PCR was performed with forward primer FBP1-F: 5′-GCTAGCATGAAGTTCTCATGT; containing *Nhe*I site and reverse primer FBP1-R: 5′-CCCGGGCTTCTTGCCCTTACT; containing the *Xma*I site without the stop codon at its C-terminal. The fragments were blunt-end inserted into phMGFP amplified by transformation into the high efficiency *Escherichia coli* Top10F'competent (Invitrogen) by the heat shock method. The plasmids were extracted and purified with QIAprep Spin Miniprep Kit (Qiagen). The Sf9 cells were seeded at a density of 5×10^5^ cells onto the cover slips in Sf9-S2 medium (PPA). The phMGFP and phMGFP-FBP1 plasmids were transfected into Sf9 cells using Transfast™ Transfection reagent (Invitrogen) according to the product instructions and cultured in a 28°C incubator. After 48 h of transfection, the cells were washed by PBS twice. The plasma membrane was stained with 2 mM DiI (Invitrogen) for 15 min at 4°C and the Sf9 cells were observed directly with the confocal laser scanning microscope.

### Localization of *Pm*Fortilin in the presence of FBP1 by immunocytochemist

Sf9 cells, at a density of 5×10^6^ cells, were grown on glass coverslips in Sf9-S2 medium (PPA), then cotransfected with phMGFP-FBP1 and pCDNA-*Pm*Fortilin using Transfast™ Transfection reagent (Invitrogen) according to the product instructions. Cells were cultured in a 28°C incubator. 48 h post-transfection, the cells were washed twice with phosphate-buffered saline (PBS) and fixed with 4% paraformaldehyde for 10 min followed by washing with 0.1% Triton x-100 in PBS for 3 min at 4°C. Fixed cells were washed again with PBS before blocking with 5% skim milk at 4°C for 1 h. After incubating, cells were washed and incubated with specific rabbit anti-*Pm*Fortilin antibody (dilution of 1∶500 in PBS) at 4°C for 16–18 h. The cells were washed 3 times with PBST and incubated with Alexa flour 647-conjugated goat anti-rabbit IgG antibody (Invitrogen, 1∶1,000 in PBS) at room temperature for 2 h. After washing 3 times with PBST, the cover glasses were mounted with 10% glycerol in PBS. The fluorescence signal was detected using a confocal laser scanning microscope (Olympus FV300).

### Yeast two-hybrid assay

The yeast two-hybrid screens were performed with the Clontech Matchmaker GAL4 Two-Hybrid System 3 (Clontech Laboratories, Inc., Mountain View, CA, USA). *Pm*Fortilin was divided into 3 fragments: *Pm*FT1 (residues 1–70), *Pm*FT2 (residues 71–120) and *Pm*FT3 (residues 121–168). Plasmids pGBKT7 (named BD vectors) were used for construction of BD-*Pm*Fortilin. The plasmids were constructed by inserting into *Eco*RI and *Sal*I sites on a BD-bait vector. Each fragment was amplified by PCR using the following primers;

BD-*Pm*FT1:

BD-*Pm*FT1-F: 5′-CGGAATTCATGAAGGTCTTCAAG,
BD-*Pm*FT1-R: 5′-GCGTCGACTCATATAACTACATCAACAC

BD-*Pm*FT2:

BD-*Pm*FT2-F: 5′-GGAATTCTATATGCGTCTGCAGGAAAC,
BD-*Pm*FT2-R: 5′-GCGTCGACTCACAAAAGGTCTGTCA

and BD-*Pm*FT3:

BD-*Pm*FT3-F: 5′-CGGAATTCAAGAAGTTCAAGGACTTGCA,
BD-*Pm*FT3-R: 5′-GCGTCGACTTATAGCTTCTCCTCTGT.

The plasmid pGADT7 (named AD vector) was used for construction of the AD-FBP1 and contained the *Bam*HI and *Xho*I sites;

AD-FBP1:

AD-FBP1-F: 5′-GGATCCCGATGAAGTTCTCATGTAAAGT,
AD-FBP1-R: 5′-CCCTCGAGTTACTTCTTGCCCTTACTAT.

All plasmids constructed were verified by sequencing. Competent yeast cells, *Saccharomyces cerevisiae* strain AH109 were co-transformed either with BD-*Pm*FT1 and AD-FBP1, BD-*Pm*FT2 and AD-FBP1, BD-*Pm*FT3 and AD-FBP1. Cells were then spread on SD selective medium lacking tryptophan, leucine, histidine and adenine (SD-TLHA). The plates were incubated at 30°C until positive colonies were visible after 2–4 days. The transformations were confirmed by PCR. The positive clones were in addition assayed by a filter lift β-galactosidase assay for LacZ activity as described in the manufacturer's protocols.

## Supporting Information

Figure S1 The ramachandran analysis of the Fortilin model from *Penaeus monodon*. The number of residues in most favored regions [A, B, L] is 108 (72.50%), the number of residues in additional allowed regions [a, b, l, p] is 33 (22.10%), the number of residues in generously allowed regions [∼a, ∼b, ∼l, ∼p] is 7 (4.70%) and one (0.70%) residue in disallowed regions. The number of non-glycine and non-proline residues is 149 (88.69%), the number of end-residues (excl. Gly and Pro) is 2 (1.19%), the number of glycine residues (shown as triangles) is 11 (6.55%) and the number of proline residues is 6 (3.57%).
(TIF)

Figure S2 The ramachandran analysis of the FBP1 model. The number of residues in most favoured regions [A, B, L] is 31 (49.20%), the number of residues in additional allowed regions [a, b, l, p] is 21 (33.30%), the number of residues in generously allowed regions [∼a, ∼b, ∼l, ∼p] is 6 (9.50%), the number of residues in disallowed regions is 5 (7.90%). The number of non-glycine and non-proline residues is 63 (67.74%), the number of end-residues (excl. Gly and Pro) is 2 (2.15%), the number of glycine residues (shown as triangles) is 3 (3.23%) and the number of proline residues is 25 (26.88%).
(TIF)

Figure S3 Multimer simulations of *Pm*Fortilin/FBP1 interaction model, based on balance mode, FBP1 as the receptor and *Pm*Fortilin as the ligand. (A) The simulation results of the FBP1 monomer combined with two *Pm*Fortilin molecules (1∶2), yields the lowest docking energy of −913.00 Kcal/mol. (B) FBP1 monomer combined with three *Pm*Fortilins (1∶3), yields the lowest docking energy of −1,244.80 Kcal/mol. (C) Two FBP1 molecules combined with two *Pm*Fortilins (2∶2), yields the lowest docking energy of −1,015.80 Kcal/mol. (D) Two FBP1 molecules and three *Pm*Fortilins (2∶3), yields the lowest docking energy of −1,015.80 Kcal/mol. The Ca^2+^-binding domain orange color and the TCTP signature 1 of the flexible loop is green.
(TIF)

Table S1 The SMART and Motif Scan analysis for *Pm*Fortilin and FBP1.
(DOCX)

Table S2 The SMART analysis result of FBP1 on the SMART database.
(DOCX)

Table S3 The 3D structure alignment of FBP1 on ASTRAL40 database (version 1.75).
(DOCX)

Table S4 List of the balance mode docking simulation of *Pm*Fortilin/FBP1.
(DOCX)

Table S5 List of the electrostatic mode docking simulation of *Pm*Fortilin/FBP1.
(DOCX)

Table S6 List of the hydrophobic mode docking simulation of *Pm*Fortilin/FBP1.
(DOCX)

Table S7 List of the VdW+Elec mode docking simulation of *Pm*Fortilin/FBP1.
(DOCX)
