# Supplementary figures and images for: Characterization of a Novel Binding Protein for Fortilin/TCTP — Component of a Defense Mechanism against Viral Infection in Penaeus monodon
Source: PLoS One. 2012 Mar 12;7(3):e33291. doi: 10.1371/journal.pone.0033291 (PMC3299765; doi:10.1371/journal.pone.0033291)

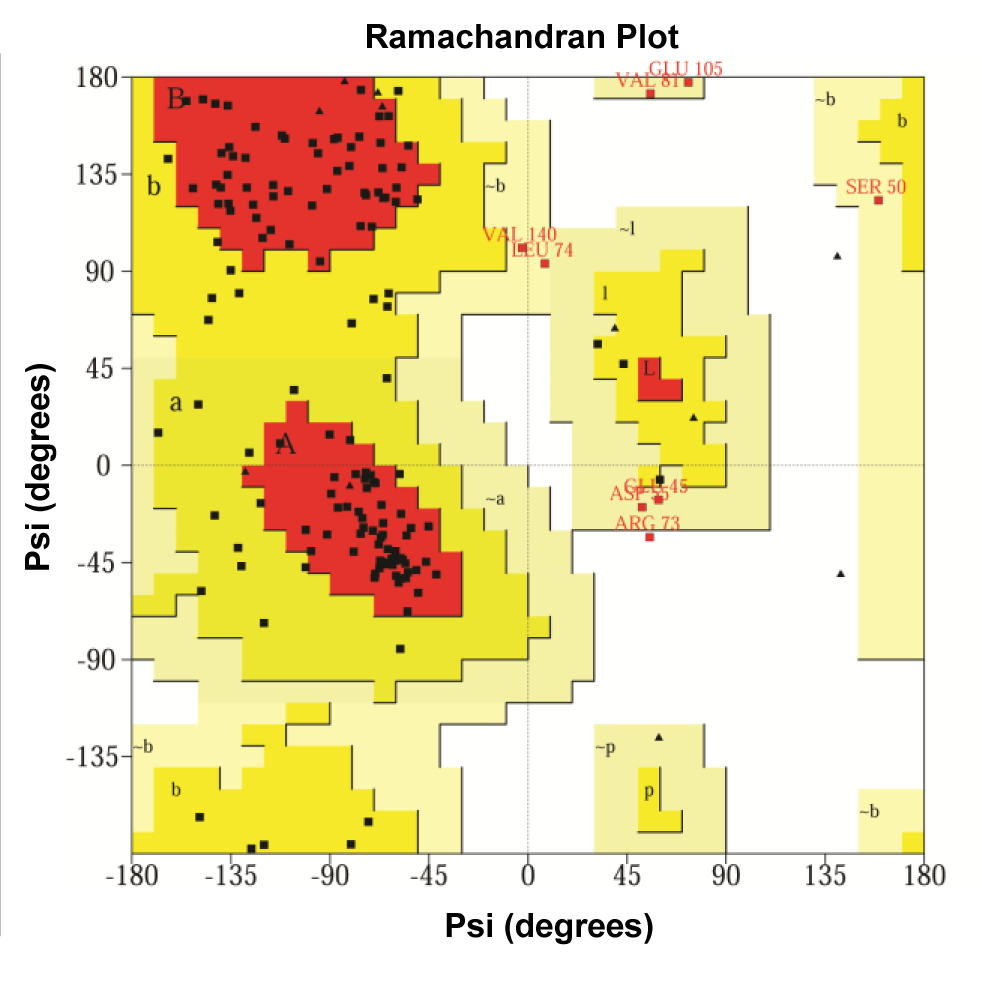

Supplement: Figure S1 — The ramachandran analysis of the Fortilin model from Penaeus monodon. The number of residues in most favored regions [A, B, L] is 108 (72.50%), the number of residues in additional allowed regions [a, b, l, p] is 33 (22.10%), the number of residues in generously allowed regions [∼a, ∼b, ∼l, ∼p] is 7 (4.70%) and one (0.70%) residue in disallowed regions. The number of non-glycine and non-proline residues is 149 (88.69%), the number of end-residues (excl. Gly and Pro) is 2 (1.19%), the number of glycine residues (shown as triangles) is 11 (6.55%) and the number of proline residues is 6 (3.57%). (TIF) [file pone.0033291.s001.tif]

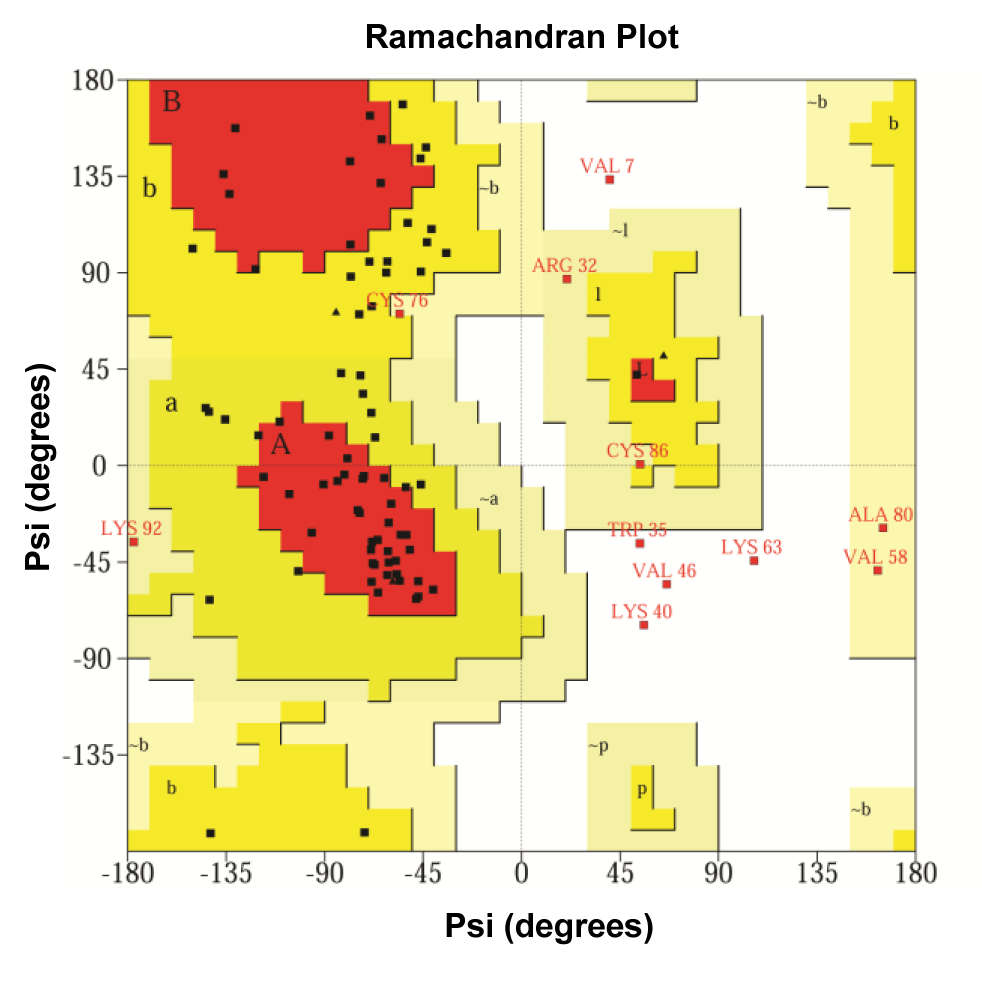

Supplement: Figure S2 — The ramachandran analysis of the FBP1 model. The number of residues in most favoured regions [A, B, L] is 31 (49.20%), the number of residues in additional allowed regions [a, b, l, p] is 21 (33.30%), the number of residues in generously allowed regions [∼a, ∼b, ∼l, ∼p] is 6 (9.50%), the number of residues in disallowed regions is 5 (7.90%). The number of non-glycine and non-proline residues is 63 (67.74%), the number of end-residues (excl. Gly and Pro) is 2 (2.15%), the number of glycine residues (shown as triangles) is 3 (3.23%) and the number of proline residues is 25 (26.88%). (TIF) [file pone.0033291.s002.tif]

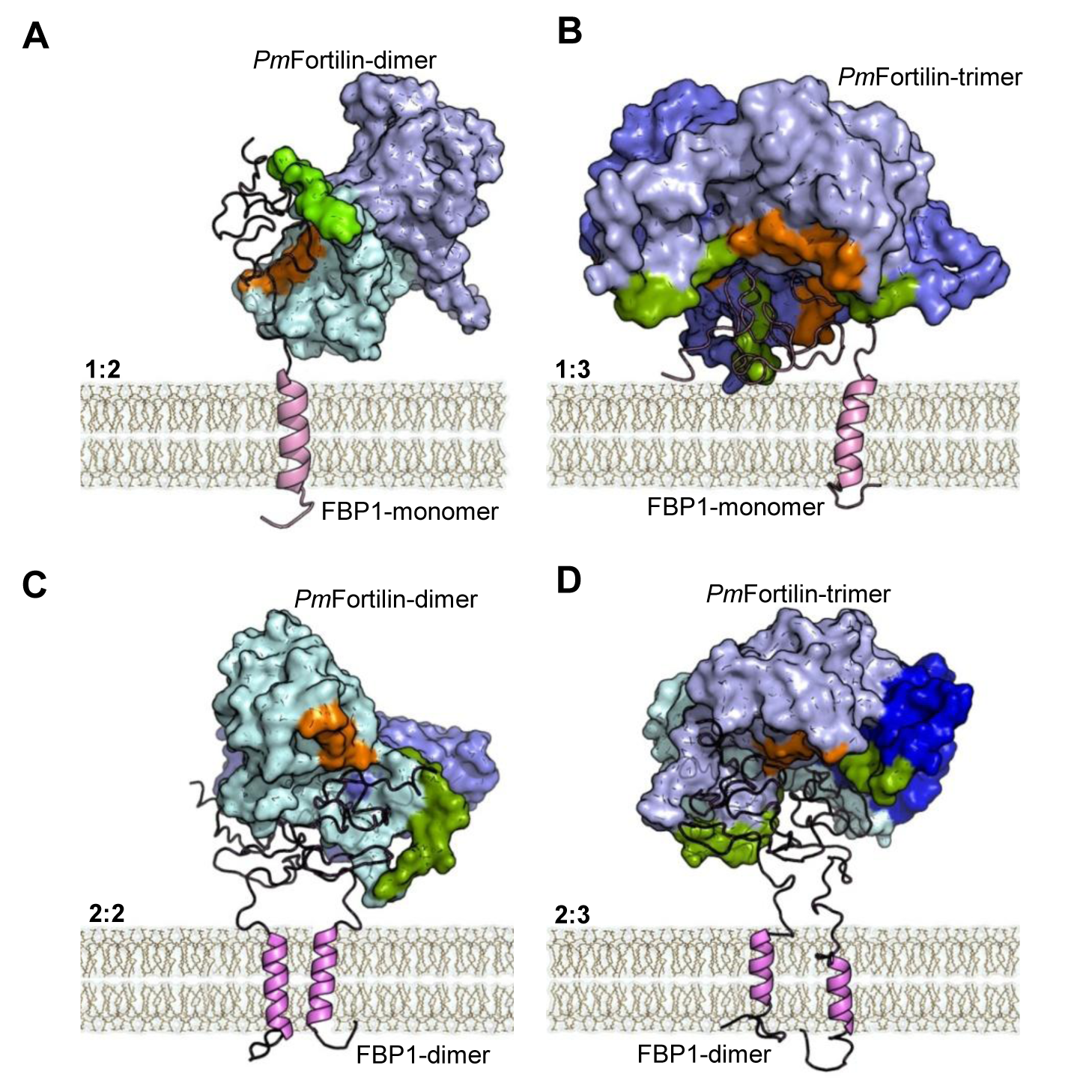

Supplement: Figure S3 — Multimer simulations of PmFortilin/FBP1 interaction model, based on balance mode, FBP1 as the receptor and PmFortilin as the ligand. (A) The simulation results of the FBP1 monomer combined with two PmFortilin molecules (1∶2), yields the lowest docking energy of −913.00 Kcal/mol. (B) FBP1 monomer combined with three PmFortilins (1∶3), yields the lowest docking energy of −1,244.80 Kcal/mol. (C) Two FBP1 molecules combined with two PmFortilins (2∶2), yields the lowest docking energy of −1,015.80 Kcal/mol. (D) Two FBP1 molecules and three PmFortilins (2∶3), yields the lowest docking energy of −1,015.80 Kcal/mol. The Ca2+-binding domain orange color and the TCTP signature 1 of the flexible loop is green. (TIF) [file pone.0033291.s003.tif]
