# Supplementary material for: Characterization of a Novel Binding Protein for Fortilin/TCTP — Component of a Defense Mechanism against Viral Infection in Penaeus monodon
Source: PLoS One. 2012 Mar 12;7(3):e33291. doi: 10.1371/journal.pone.0033291 (PMC3299765; doi:10.1371/journal.pone.0033291)
Supplement: Table S1 — The SMART and Motif Scan analysis for PmFortilin and FBP1. (DOCX) [file pone.0033291.s004.docx]

**Table S1.** The SMART and Motif Scan analysis for *Pm*Fortilin and FBP1**.**

| **Proteins** | **Motif information** | **Amino acid residues** | **Analysis Tools** |
| --- | --- | --- | --- |
| *Pm*Fortilin | N–glycosylation site | 34–37 | Motif Scan |
|  | Casein kinase II phosphorylation site | 9–12, 36–39, 50–53, 64–67 | Motif Scan |
|  | N–myristoylation site | 57–62, 102–107 | Motif Scan |
|  | Protein kinase C phosphorylation site | 17–19 | Motif Scan |
|  | Translationally controlled tumor protein signature 1 | 45–55 | Motif Scan |
|  | Translationally controlled tumor protein signature 2 | 123–145 | Motif Scan |
| FBP1 | Amidation site | 90–93 | Motif Scan |
|  | Protein kinase C phosphorylation site | 4–6 | Motif Scan |
|  | Cysteine–rich region profile | 5–86 | Motif Scan |
|  | Proline–rich region profile | 27–87 | Motif Scan |
|  | Zinc finger A20–type profile | 36–71 | Motif Scan |
|  | Signal peptide | 1–24 | SMART |
|  | Transmembrane segment | 7–26 | SMART |
|  | Low complexity | 27–48, 59–87 | SMART |
