# Supplementary material for: Characterization of a Novel Binding Protein for Fortilin/TCTP — Component of a Defense Mechanism against Viral Infection in Penaeus monodon
Source: PLoS One. 2012 Mar 12;7(3):e33291. doi: 10.1371/journal.pone.0033291 (PMC3299765; doi:10.1371/journal.pone.0033291)
Supplement: Table S2 — The SMART analysis result of FBP1 on the SMART database. (DOCX) [file pone.0033291.s005.docx]

**Table S2.** The SMART analysis result of FBP1 on the SMART database.

|  | FBP1 Positions | |  |  |
| --- | --- | --- | --- | --- |
| Genes | Begin | End | E–value^1^ | FBP1 amino acids |
| *ZnF_C4* | 2 | 45 | 7.76E+02 | KFSCKVCLLGFCALVIICAVAEATSPPGPFRCPWRPPCKKCRPR |
| [*Amb_V_allergen*](http://smart.embl-heidelberg.de/smart/do_annotation.pl?DOMAIN=Amb_V_allergen&TYPE=SMART&START=10&END=52&LENGTH=42&E_VALUE=49235.285233826&BLAST=LGFCALVIICAVAEATSPPGPFRCPWRPPCKKCRPRVCPAIAC) | 10 | 52 | 4.92E+04 | LGFCALVIICAVAEATSPPGPFRCPWRPPCKKCRPRVCPAIAC |
| *PHD* | 4 | 40 | 4.48E+02 | SCKVCLLGFCALVIICAVAEASPPGPFRCPWRPPCK |
| *PSI* | 18 | 60 | 2.77E+03 | ICAVAEATSPPGPFRCPWRPPCKKCRPRVCPAIACPKYEDVCP |
| [*PostSET*](http://smart.embl-heidelberg.de/smart/do_annotation.pl?DOMAIN=PostSET&TYPE=SMART&START=72&END=88&LENGTH=16&E_VALUE=2506.41401414377&BLAST=PPPPCNCPAPVCPPCPY) | 72 | 88 | 2.51E+03 | PPPPCNCPAPVCPPCPY |
| *TNFR* | 19 | 47 | 2.32E+03 | CAVAEATSPPGPFRCPWRPPCKKCRPRVC |
| *ZnF_A20* | 39 | 64 | 1.24E+03 | CKKCRPRVCPAIACPKYEDVCPPCKP |

The list of genes was ranking by the E-value score. **^1^**The E-values of the SMART analysis parameters default by HMMER searches of the SMART database including the outlier homologues and homologues of known structure, PFAM domains, signal peptides, internal repeats and intrinsic protein disorder database.
