# Supplementary material for: Characterization of a Novel Binding Protein for Fortilin/TCTP — Component of a Defense Mechanism against Viral Infection in Penaeus monodon
Source: PLoS One. 2012 Mar 12;7(3):e33291. doi: 10.1371/journal.pone.0033291 (PMC3299765; doi:10.1371/journal.pone.0033291)
Supplement: Table S3 — The 3D structure alignment of FBP1 on ASTRAL40 database (version 1.75). (DOCX) [file pone.0033291.s006.docx]

**Table S3.** The 3D structure alignment of FBP1 on ASTRAL40 database (version 1.75).

| SCOP classification^1^ | | Amino acid residues | |  |  |  |  |
| --- | --- | --- | --- | --- | --- | --- | --- |
| PDB | SCOP proteins | SCOP | FBP1 | Equ.^2^ (%) | Resi.^3^ | SSEs^4^ | RMSD^5^ (Å) |
| 1NT4 | Glucose–1–phosphatase | 254 –280 | 1–27 | 47 | 44 | 4 | 3.55 |
|  |  | 282–287 | 29–34 |  |  |  |  |
|  |  | 14–15 | 49–50 |  |  |  |  |
|  |  | 24–32 | 84–92 |  |  |  |  |
| 1AZS | Adenylyl cyclase VC1, domain C1a | 76– 96 | 3–23 | 46 | 43 | 4 | 2.6 |
|  |  | 105–107 | 24–26 |  |  |  |  |
|  |  | 146–153 | 27–34 |  |  |  |  |
|  |  | 159–169 | 44–54 |  |  |  |  |
| 1RYP | Proteasome beta subunit (catalytic) | 71–102 | 3–34 | 46 | 43 | 3 | 3.17 |
|  |  | 104 –114 | 41–51 |  |  |  |  |
| 1IUG | Aaspartate aminotransferase subgroup IV | 252–270 | 4–27 | 46 | 43 | 4 | 3.26 |
|  |  | 319–327 | 30–38 |  |  |  |  |
|  |  | 133–134 | 49–50 |  |  |  |  |
|  |  | 185–197 | 78–90 |  |  |  |  |
| 1RYP | Proteasome alpha subunit (non–catalytic) | 104–121 | 5–22 | 45 | 42 | 4 | 2.72 |
|  |  | 126–135 | 23–32 |  |  |  |  |
|  |  | 139–148 | 41–50 |  |  |  |  |
|  |  | 216–219 | 85–88 |  |  |  |  |
| 2PHD | Gentisate 1, 2–dioxygenase | 42–60 | 6–24 | 45 | 42 | 4 | 2.94 |
|  |  | 63–73 | 25–35 |  |  |  |  |
|  |  | 155–159 | 49–53 |  |  |  |  |
|  |  | 311–317 | 82–88 |  |  |  |  |
| 1RYP | Proteasome alpha subunit (non–catalytic) | 100–119 | 4–23 | 45 | 42 | 3 | 3.19 |
|  |  | 124–134 | 24–34 |  |  |  |  |
|  |  | 135–145 | 41–51 |  |  |  |  |
| 1FSG | Hypoxanthine–guanine–xanthine PRTase | 154–174 | 8–28 | 45 | 42 | 4 | 3.22 |
|  |  | 187–195 | 48–56 |  |  |  |  |
|  |  | 213–224 | 78–89 |  |  |  |  |
| 1DP4 | Atrial natriuretic peptide receptor A | 307–330 | 2–25 | 45 | 42 | 4 | 3.48 |
|  |  | 367–375 | 27–35 |  |  |  |  |
|  |  | 236–239 | 83–86 |  |  |  |  |
|  |  | 218–222 | 88–92 |  |  |  |  |

The list of SCOP classified proteins was cut–off and ranking by the value of equivalence ≥ 45%. **^1^**SCOP classification is a string containing the classified SCOP superfamily name for a given protein structure. **^2^**The equivalences (Equ.) are the fraction of aligned residues, including coiled regions with regard to the smaller of both molecules. By default, the best alignment is defined as the structure alignment with the largest fraction of aligned residues. The RMSD is kept below ~4Å. **^3^**The residues (Resi.) are the number of aligned residues. By default the entries of the structure alignment database browser are sorted in ascending order, according to this quantity. **^4^**The SSEs are the number of aligned SSE pairs between the two protein structures. Only helices and sheets are considered here. However the total number of aligned residues includes coiled regions also. The alignment is enlarged in a gapless manner based on a valid SSE assignment. **^5^**RMSD is the C-alpha root mean square deviation of aligned residues.
