# Supplementary material for: Characterization of a Novel Binding Protein for Fortilin/TCTP — Component of a Defense Mechanism against Viral Infection in Penaeus monodon
Source: PLoS One. 2012 Mar 12;7(3):e33291. doi: 10.1371/journal.pone.0033291 (PMC3299765; doi:10.1371/journal.pone.0033291)
Supplement: Table S4 — List of the balance mode docking simulation of PmFortilin/FBP1. (DOCX) [file pone.0033291.s007.docx]

**Table S4.** List of the balance mode docking simulation of *Pm*Fortilin/FBP1.

|  |  | Balance mode weight energy scores | |  |
| --- | --- | --- | --- | --- |
| Ranking^1^ | Cluster (members) | Center energy (Kcal/mol) | Lowest energy (Kcal/mol) | Binding conformations |
| 1 | 0 (177) | –793.80 | –993.40 | Conformation A |
| 2 | 2 (95) | –780.90 | –958.10 | Conformation A |
| 3 | 4 (50) | –774.30 | –927.80 | Conformation A |
| 4 | 3 (67) | –797.70 | –925.40 | Conformation A |
| 5 | 15 (22) | –764.00 | –920.30 | Conformation B |
| 6 | 1 (97) | –820.60 | –917.30 | Conformation B |
| 7 | 14 (24) | –915.90 | –915.90 | Conformation B |
| 8 | 6 (48) | –896.00 | –896.00 | Conformation A |
| 9 | 18 (17) | –769.10 | –891.50 | Conformation A |
| 10 | 5 (48) | –768.40 | –891.00 | Conformation A |
| 11 | 9 (29) | –845.30 | –883.10 | Conformation A |
| 12 | 11 (27) | –801.20 | –878.70 | Conformation A |
| 13 | 7 (37) | –852.10 | –869.20 | Conformation B |
| 14 | 19 (16) | –863.60 | –863.60 | Conformation A |
| 15 | 12 (26) | –862.20 | –862.20 | Conformation A |
| 16 | 16 (21) | –856.30 | –856.30 | Conformation B |
| 17 | 21 (13) | –811.60 | –838.80 | Conformation A |
| 18 | 8 (31) | –838.00 | –838.00 | Conformation B |
| 19 | 10 (28) | –832.40 | –832.40 | Conformation B |
| 20 | 17 (17) | –780.50 | –831.20 | Conformation A |
| 21 | 22 (10) | –765.90 | –819.40 | Conformation A |
| 22 | 23 (5) | –786.80 | –809.40 | Conformation A |
| 23 | 13 (24) | –760.50 | –806.00 | Conformation A |
| 24 | 20 (14) | –780.90 | –796.00 | Conformation A |
| 25 | 24 (1) | –764.20 | –764.20 | Conformation A |
|  | Average | –811.29 | –871.41 |  |

^1^The ranking positions were ordering by the lowest energy of docking simulation scored.
