# Supplementary material for: Characterization of a Novel Binding Protein for Fortilin/TCTP — Component of a Defense Mechanism against Viral Infection in Penaeus monodon
Source: PLoS One. 2012 Mar 12;7(3):e33291. doi: 10.1371/journal.pone.0033291 (PMC3299765; doi:10.1371/journal.pone.0033291)
Supplement: Table S5 — List of the electrostatic mode docking simulation of PmFortilin/FBP1. (DOCX) [file pone.0033291.s008.docx]

Table S5. List of the electrostatic mode docking simulation of *Pm*Fortilin/FBP1.

|  |  | Electrostatic mode weight energy scores | |  |
| --- | --- | --- | --- | --- |
| Ranking^1^ | Cluster (members) | Center energy (Kcal/mol) | Lowest energy (Kcal/mol) | Binding conformations |
| 1 | 2 (123) | –1,054.30 | –1,262.30 | Conformation A |
| 2 | 1 (158) | –1,030.10 | –1,246.30 | Conformation A |
| 3 | 3 (80) | –986.40 | –1,238.50 | Conformation A |
| 4 | 0 (159) | –1,100.90 | –1,229.70 | Conformation A |
| 5 | 10 (28) | –1,082.50 | –1,222.20 | Conformation B |
| 6 | 7 (43) | –1,012.00 | –1,178.00 | Conformation A |
| 7 | 5 (49) | –984.00 | –1,163.50 | Conformation A |
| 8 | 15 (18) | –993.30 | –1,155.30 | Conformation A |
| 9 | 8 (41) | –1,140.90 | –1,140.90 | Conformation B |
| 10 | 6 (44) | –998.00 | –1,115.90 | Conformation B |
| 11 | 21 (3) | –1,029.30 | –1,112.80 | Conformation A |
| 12 | 9 (33) | –1,103.10 | –1,103.10 | Conformation B |
| 13 | 19 (10) | –1,102.30 | –1,102.30 | Conformation B |
| 14 | 11 (25) | –1,005.60 | –1,099.00 | Conformation B |
| 15 | 4 (53) | –1,013.90 | –1,072.00 | Conformation A |
| 16 | 16 (12) | –1,052.30 | –1,064.20 | Conformation A |
| 17 | 17 (12) | –1,052.30 | –1,052.30 | Conformation A |
| 18 | 14 (20) | –1,029.70 | –1,045.80 | Conformation A |
| 19 | 13 (21) | –985.50 | –1,045.20 | Conformation A |
| 20 | 12 (23) | –1,011.70 | –1,040.40 | Conformation A |
| 21 | 18 (11) | –1,039.70 | –1,039.70 | Conformation A |
| 22 | 22 (2) | –987.60 | –992.50 | Conformation A |
| 23 | 20 (5) | –987.60 | –990.60 | Conformation A |
|  | Average | –1,034.04 | –1,117.93 |  |

^1^The ranking positions were ordering by the lowest energy of docking simulation scored.
