# Supplementary material for: Characterization of a Novel Binding Protein for Fortilin/TCTP — Component of a Defense Mechanism against Viral Infection in Penaeus monodon
Source: PLoS One. 2012 Mar 12;7(3):e33291. doi: 10.1371/journal.pone.0033291 (PMC3299765; doi:10.1371/journal.pone.0033291)
Supplement: Table S6 — List of the hydrophobic mode docking simulation of PmFortilin/FBP1. (DOCX) [file pone.0033291.s009.docx]

**Table S6.** List of the hydrophobic mode docking simulation of *Pm*Fortilin/FBP1.

|  |  | Hydrophobic mode weight energy scores | |  |
| --- | --- | --- | --- | --- |
| Ranking^1^ | Cluster (members) | Center energy (Kcal/mol) | Lowest energy (Kcal/mol) | Binding conformations |
| 1 | 0 (191) | –950.90 | –1,230.50 | Conformation B |
| 2 | 1 (76) | –970.90 | –1,202.70 | Conformation B |
| 3 | 5 (51) | –917.50 | –1,151.10 | Conformation A |
| 4 | 7 (44) | –988.30 | –1,141.00 | Conformation B |
| 5 | 2 (74) | –939.10 | –1,114.50 | Conformation B |
| 6 | 4 (64) | –939.70 | –1,106.00 | Conformation A |
| 7 | 6 (44) | –931.30 | –1,070.40 | Conformation B |
| 8 | 11 (37) | –957.90 | –1,064.70 | Conformation B |
| 9 | 8 (42) | –926.10 | –1,045.30 | Conformation A |
| 10 | 12 (36) | –1,040.00 | –1,040.00 | Conformation A |
| 11 | 19 (13) | –912.20 | –1,015.90 | Conformation B |
| 12 | 13 (22) | –908.90 | –1,013.50 | Conformation B |
| 13 | 22 (4) | –898.20 | –1,012.10 | Conformation A |
| 14 | 16 (16) | –903.60 | –1,011.10 | Conformation B |
| 15 | 3 (70) | –948.10 | –999.30 | Conformation B |
| 16 | 9 (41) | –999.30 | –999.30 | Conformation B |
| 17 | 14 (20) | –997.10 | –997.10 | Conformation B |
| 18 | 15 (18) | –992.90 | –992.90 | Conformation B |
| 19 | 18 (13) | –903.90 | –988.50 | Conformation B |
| 20 | 21 (9) | –905.80 | –974.50 | Conformation B |
| 21 | 10 (37) | –900.60 | –966.50 | Conformation B |
| 22 | 17 (14) | –898.30 | –963.50 | Conformation B |
| 23 | 20 (11) | –898.90 | –951.50 | Conformation A |
|  | Average | –940.41 | –1,045.73 |  |

^1^The ranking positions were ordering by the lowest energy of docking simulation scored.
