# Supplementary material for: Characterization of a Novel Binding Protein for Fortilin/TCTP — Component of a Defense Mechanism against Viral Infection in Penaeus monodon
Source: PLoS One. 2012 Mar 12;7(3):e33291. doi: 10.1371/journal.pone.0033291 (PMC3299765; doi:10.1371/journal.pone.0033291)
Supplement: Table S7 — List of the VdW+Elec mode docking simulation of PmFortilin/FBP1. (DOCX) [file pone.0033291.s010.docx]

**Table S7.** List of the VdW+Elec mode docking simulation of *Pm*Fortilin/FBP1.

|  |  | VdW+Elec mode weight energy scores | |  |
| --- | --- | --- | --- | --- |
| Ranking^1^ | Cluster (members) | Center energy (Kcal/mol) | Lowest energy (Kcal/mol) | Binding conformations |
| 1 | 0 (173) | –311.90 | –387.90 | Conformation A |
| 2 | 2 (76) | –315.60 | –369.50 | Conformation A |
| 3 | 1 (151) | –317.50 | –360.50 | Conformation A |
| 4 | 10 (33) | –326.40 | –356.40 | Conformation A |
| 5 | 4 (51) | –355.80 | –355.80 | Conformation A |
| 6 | 5 (45) | –304.40 | –353.70 | Conformation A |
| 7 | 20 (12) | –307.20 | –352.90 | Conformation A |
| 8 | 8 (42) | –305.00 | –344.10 | Conformation A |
| 9 | 3 (53) | –314.40 | –342.30 | Conformation A |
| 10 | 7 (44) | –319.10 | –341.90 | Conformation A |
| 11 | 6 (44) | –334.80 | –340.10 | Conformation A |
| 12 | 16 (22) | –311.30 | –337.60 | Conformation A |
| 13 | 21 (12) | –333.20 | –336.10 | Conformation A |
| 14 | 14 (26) | –301.70 | –335.80 | Conformation A |
| 15 | 11 (32) | –318.00 | –335.60 | Conformation B |
| 16 | 12 (29) | –307.30 | –331.10 | Conformation A |
| 17 | 9 (35) | –306.70 | –328.00 | Conformation A |
| 18 | 13 (26) | –304.00 | –326.10 | Conformation A |
| 19 | 15 (23) | –307.00 | –322.50 | Conformation A |
| 20 | 18 (13) | –308.10 | –321.30 | Conformation A |
| 21 | 19 (13) | –317.60 | –320.10 | Conformation A |
| 22 | 22 (2) | –302.30 | –316.30 | Conformation A |
| 23 | 17 (15) | –315.70 | –315.70 | Conformation A |
|  | Average | –315.00 | –340.49 |  |

^1^The ranking positions were ordering by the lowest energy of docking simulation scored.
